# Developing a similarity searching module for patient safety event reporting system using semantic similarity measures

**DOI:** 10.1186/s12911-017-0467-8

**Published:** 2017-07-05

**Authors:** Hong Kang, Yang Gong

**Affiliations:** School of Biomedical Informatics, the University of Texas Health Science Center at Houston, 7000 Fannin St., Houston, TX 77030 USA

**Keywords:** Patient safety, Medical errors, Medical informatics

## Abstract

**Background:**

The most important knowledge in the field of patient safety is regarding the prevention and reduction of patient safety events (PSE) during treatment and care. The similarities and patterns among the events may otherwise go unnoticed if they are not properly reported and analyzed. There is an urgent need for developing a PSE reporting system that can dynamically measure the similarities of the events and thus promote event analysis and learning effect.

**Methods:**

In this study, three prevailing algorithms of semantic similarity were implemented to measure the similarities of the 366 PSE annotated by the taxonomy of The Agency for Healthcare Research and Quality (AHRQ). The performance of each algorithm was then evaluated by a group of domain experts based on a 4-point Likert scale. The consistency between the scales of the algorithms and experts was measured and compared with the scales randomly assigned. The similarity algorithms and scores, as a self-learning and self-updating module, were then integrated into the system.

**Results:**

The result shows that the similarity scores reflect a high consistency with the experts’ review than those randomly assigned. Moreover, incorporating the algorithms into our reporting system enables a mechanism to learn and update based upon PSE similarity.

**Conclusion:**

In conclusion, integrating semantic similarity algorithms into a PSE reporting system can help us learn from previous events and provide timely knowledge support to the reporters. With the knowledge base in the PSE domain, the new generation reporting system holds promise in educating healthcare providers and preventing the recurrence and serious consequences of PSE.

## Background

An effective way to learn from patient safety events (PSE) is through reporting system, where events are collected in a properly structured format [1]. With the accumulation of the event reports, such a system will turn into a knowledge base of PSE repository which could generate common solutions for cases under investigation [2]. In order to achieve this goal, researchers must meet two essential challenges: 1) how to define the structured format of PSE; and 2) how to measure the similarity between two PSE. The Common Formats (CF) released by The Agency for Healthcare Research and Quality (AHRQ) [3] and the International Classification of Patient Safety (ICPS) released by the World Health Organization (WHO) [4] defined the types and categories for PSE, which are widely accepted and commonly used in patient safety community. However, neither CF nor ICPS can provide PSE reports comprehensive profiles for comparison purpose which is the foundation of learning. Researchers are striving to develop new description approaches for PSE reports such as an ontology in the PSE domain which could better serve the PSE reporting and comparing. By annotating all PSE reports to the same ontology, the comparison between two PSE reports could be technically processed through semantic similarity measure as a function that, given two sets of terms annotating two entities, returns a numerical value reflecting the closeness in meaning between the two [5].

As the advent of big data era, semantic similarity algorithms have been generally applied in many fields, such as bioinformatics [5–7], geoinformatics [8], linguistics [9, 10] and natural language processing (NLP) [11, 12]. Semantic similarity assesses the degree of relatedness between two entities by the similarity in meaning of their annotations. Basically, there are two types of semantic similarity approaches when comparing terms, edge-based and node-based. Edge-based approaches are based on counting the number of edges in the graph path between two terms [13], for instance, the shortest path or the average of all paths. Correspondingly, node-based approaches focus on comparing the properties of the terms themselves, their ancestors or descendants. Information content (IC), a typical node-based approach, gives a measure of information to every term and regards the information as an important parameter when comparing different annotated entities. Edge-based and node-based approaches are intended to score the similarity between two terms, and must be extended to compare sets of terms such as gene products and PSE reports. Pairwise and group-wise approaches are the two types of strategies applicable for the comparison of term sets. Every term in the direct annotation set A is compared against every term in the direct annotation set B in pairwise approaches, then the semantic similarity is considered by every pairwise combination of terms from the two sets (average, the maximum, or sum) or only the best-matching pair for each term. Group-wise approaches calculate the similarity directly by set, graph, or vector. Set approaches are not widely used since they only consider the direct annotations that would lose a lot of information; based on set similarity techniques, graph approaches represent entities as the subgraphs of the whole annotations and calculate the similarity using graph matching techniques; vector approaches compact the information in vector space (VS) as binary fingerprints which are more convenient for comparison.

With a main focus on investigating similarity in molecular biology, the Gene Ontology (GO) [14] is the most common ontology widely adopted by the life sciences community, which enables the comparison among gene products at the functional level. Numerous researches have demonstrated that the functional relatedness between gene products with GO annotations can be well measured by semantic similarity algorithms [5, 15–19], which demonstrate major significance for gene function studies. AHRQ PSNet (Patient Safety Network) taxonomy [20], in contrast, is also imperative for understanding the meaning of patient safety and underlying concepts relate to the existing safety and quality frameworks commonly used in healthcare [21], and for presenting an opportunity for healthcare providers to learn from the previous events. In the patient safety community, there is an urgent need for an approach to comparing PSE and offering potential solutions based on the compared cases. Intuitively, the form of event data appears similar to that of GO, since a number of taxonomies have been designed for labeling cases through ontology annotations. Accordingly, the methods that work effectively to compare GO products might be feasible when identifying similarities in PSE. However, to our best knowledge, the semantic similarity algorithms have never been adopted and assessed by using patient safety data.

In this study, detailed comparisons were made between GO and the AHRQ PSNet taxonomy from multiple perspectives, based on which we reviewed the semantic similarity measures and analyzed their applicability to the AHRQ Morbidity and Mortality Rounds on the Web (WebM&M) database [20]. WebM&M, the only publicly accessible patient safety database with annotated event reports, makes it possible to compare the reports by applying the semantic similarity measures. A workflow about how to process and assess the semantic similarity measures on WebM&M data was proposed. According to the workflow, several preliminary results were raised for further discussion.

## Methods

### Dataset comparison

To ensure AHRQ PSNet taxonomy has the potentials to represent PSE features and to support PSE similarity measurement, we compared the characteristics presented in GO against those in AHRQ PSNet taxonomy from five perspectives: 1) stage of development; 2) complexity and independence; 3) quality and maintenance; 4) assessment of similarity; 5) application value of similarity study.

### Semantic similarity algorithms

We reviewed the key literature on semantic similarity measures in the field of GO through which we identified and scrutinized diverse semantic similarity approaches according the characteristics of WebM&M data. Using Pesquita’s work [5] as the primary reference, we chose three prevailing approaches from each typical type of semantic similarity as the potential assessment candidates. The approaches were applied to measure the PSE similarity by calculating a similarity score based on their annotations on the AHRQ PSNet taxonomy.

### Expert review

In order to assess the performance of the semantic similarity algorithms, three experts who hold MD degrees and have work experiences in clinical settings participated in the expert review. They are also familiar with patient safety data and the process of PSE reporting. The experts reviewed and judged the degree of relevancy between query case and every other cases through a 4-point Likert scale [22] which contains 1-irrelevant, 2-somewhat irrelevant, 3-relevant, and 4-highly relevant. After the experts completed the review, two rounds of discussion were conducted to provide a final review result. If an agreement was not reached to certain case, the case would be labeled by a majority. The final expert result was treated as a gold standard. Any case that was labeled as either 1 or 2 by both expert and algorithm was regarded as an “agreement” and judged as being irrelevant to the query case; conversely, the ones that were labeled as either 3 or 4 by expert and algorithm were also regarded as “agreements” but classified as being relevant to the query. The agreement ratio between final expert review and algorithm (sample agreement ratio) was calculated by dividing the numbers of agreement cases by the number of total cases. Then we randomly labeled the same group of cases for 10,000 times and calculated the agreement ratios respectively (random agreement ratios). One sample t-test was adopted to examine the mean difference between the sample agreement ratio and the random agreement ratios mean (power analysis).

## Results

### Comparison between GO and WebM&M with AHRQ PSNet taxonomy

#### Stage of development

To date, GO has been the most widely adopted knowledge database in the life sciences community for comparing gene products at the functional level since 1998. GO defines commonly accepted ontology and provides a schema for representing gene product function in the cellular context. The GO project has developed formal ontologies that represent over 40,000 biological concepts, which are constantly being revised to reflect new discoveries. WebM&M, an online journal and forum on patient safety and healthcare quality, features expert analysis of anonymously reported PSE. Since February 2003, WebM&M has accumulated 366 cases with annotations mapping to a 219-concept taxonomy across six axes (AHRQ PSNet taxonomy). Apparently, WebM&M has much fewer entities and a less complicated ontology/taxonomy comparing to GO. To our best knowledge, little research on PSE similarity has been conducted thus far.

#### Complexity and Independence

The structure of GO is typical directed acyclic graphs (DAGs), and each term in GO is assigned to one of the three independent root ontologies: molecular function, biological process and cellular component. Although the six axes of AHRQ PSNet taxonomy are not independent, the data structure of AHRQ PSNet taxonomy is much simpler than that of GO since it has fewer terms and lower complexity.

#### Quality and maintenance

A consortium of GO [23] is responsible for developing and maintaining GO databases as well as the tools that support the creation, maintenance, and use of all the information. The consortium ensures the high quality of GO. The situation of WebM&M is different because all the annotations are based on voluntarily submissions. Although cases in WebM&M are well scrutinized by the experts in patient safety, keeping the consistency of annotation may be difficult due to different understanding among diverse expertise across the healthcare domains.

#### Assessment of similarity

There is an increasing trend in defining functional relatedness through semantic similarity of genes and GO annotations. One reason is that the performance of similarity algorithms on GO is much easier to be assessed, since there are plenty of experimental methods providing real similarity measures for gene products which serve as references in the assessment procedure. However, the assessment of similarity algorithms on WebM&M data is much more challenging since there is still no widely accepted method which can supply a real similarity measure between two PSE.

#### Application value of similarity study

GO provides rich information and a convenient way to study gene functional similarity, which has been successfully used in various aspects including predicting gene functional associations [24], homology analysis [25], assessing target gene functions [26], and predicting subcellular localization [27]. In the patient safety field, the essential purpose of establishing computerized system for PSE reporting is to acquire experiences from previous cases, find solutions for new cases, and reduce the probability of recurrence. Therefore, finding an approach of measuring and assessing the similarity between PSE is considered the primary goal of learning from the PSE reports.

In summary, AHRQ PSNet taxonomy still has room for improvement comparing to the GO which is already a mature product in molecular biology. However, as the only hierarchical feature structure for PSE reports, PSNet taxonomy has the potentials to represent PSE features and to support PSE similarity measurement.

#### The applicability analysis for semantic similarity methods on WebM&M data

Researchers have suggested that the semantic similarity of the GO annotations of gene can serve as a proxy for functional relatedness [5]. However, whether these approaches or which of them are applicable for PSE remains unclear. Aiming to narrow the searching scope, we summarized the pros and cons for all the above mentioned semantic similarity approaches and their typical applications in biomedical researches, as shown in Table 1. Considering the characteristics of the WebM&M data, three approaches (IC model, normalized term overlap model and VS model) were finally involved in our study.

**Table 1:** Summary of semantic similarity approaches

Measures for terms				
Approach	Techniques	Algorithms		Pros & Cons
Edge-based	Distance; Common path	[16, 35–37]		Pros: intuitive, easy to perform. Cons: edge-based approaches assume all the nodes and edges are uniformly distributed and treat them who are in the same depth equally, which is not applicable for real data.
Node-based	MICA^a^; DCA^b^	[18, 28, 38–41]		Pros: node-based approaches measure the terms independent of their depth in the ontology. Cons: the common used term would make more contribution when calculating the similarity.
Measures for sets of terms				
Approach	Techniques	Algorithms		Pros & Cons
Pairwise	All pairs	[42]		Pros: the contributions from every pair of terms are concerned. Cons: over-reliance on the quality of data; time-consuming
	Best Pairs	[16, 18, 19, 39, 43]		
Group-wise	Set-based	Not common		Pros: group-wise approaches compare term combinations from a macro view instead of relying on integrating similarity between individual terms; time-saving. Cons: excessive choices could be a trouble.
	Graph-based	[15, 44–50]		
	Vector-based	[51, 52]		

^a^MICA = the most informative common ancestor

^b^DCA = the disjoint common ancestor

#### Information Content (IC)

As a classic node-based approach, IC gives a measure on how specific and informative a term is. Towards PSE, it assumes that a term with higher probability of occurrence may contribute less when measuring the similarity. In this study, the pairwise strategy which calculated the similarity for all pairs of terms and assessed them with average score was adopted. And we used Lin’s measure of similarity [28] which accounts the IC values for each of term *t*_*1*_ and *t*_*2*_ in addition to the lowest ancestor shared between the two terms.

#### Normalized Term Overlap (NTO)

NTO [15] considers the set of all direct annotations and all of their associated parent terms. Theoretically, NTO might be applicable if the taxonomy is well defined and the reports are well annotated. The only concern is that the depth of AHRQ PSNet taxonomy may be not deep enough to ensure the expected performance of NTO. However, to figure out the applicability of this typical graph-based group-wise approach, we also enrolled this method to our assessment.

#### Vector Space (VS)

VS compacts the annotations of a set of terms into a binary vector which is more comparable because the model is based on linear algebra with lots of mature algorithms which can measure similarity, such as cosine measure [29]. Similar to IC, a variation of VS approach has been used in ontology-based similarity. The approach generates a weight for each term based on the frequency of its occurrence in the corpus, and then replaces the non-zero values in the binary vector with these weights. As the WebM&M cases are well annotated in an ontological structure, VS measure, a vector based group-wise approach, may be potentially applicable to measure the similarity between WebM&M cases.

### Workflow for semantic similarity analysis on WebM&M data

#### Data collection and management

Each WebM&M report contains three parts of information (summary, commentary and references) and has been annotated by AHRQ PSNet taxonomy with six perspectives and 219 totally terms. All the reports and annotations were extracted from WebM&M and managed in our local MySQL database server.

#### Algorithm implementation

For each approach of IC, NTO and VS, we initialized the weights of the six perspectives equally to calculate the similarity score between every pair of PSE. These weights and the similarity matric would be optimized dynamically all the times based on the feedback or assessment from domain experts and end users.

#### Expert review

Expert review introduced in the Methods session were carried out to create the gold standard and assess the performance of the PSE similarity searching model.

#### Agreement analysis

In order to investigate the agreement between the results provided by an expert and a certain semantic similarity approach, we firstly labeled the same amount of cases which were ranked by similarity scores with a 4-point Likert scale according to the same distribution ratio of the scales rated by the expert. For instance, if the expert labeled three cases with 1-irrelevant, four cases with 2-somewhat irrelevant, six cases with 3-relevant, and seven cases with 4-highly relevant, we would label three cases with the lowest similarity scores as label 1, then label the 4th to 7th lowest ones as label 2, and so on. An agreement ratio was calculated to represent the consistency between each pair of expert and semantic similarity approach.

#### Statistical test

The performance of semantic similarity approaches was assessed by testing the mean difference of the agreement ratios between the random group and the gold standard.

#### User feedback

The semantic similarity module was embedded into our voluntary PSE reporting system which allows users to provide feedback by clicking a user feedback button to decide whether they agree or disagree with certain case with high similarity against the query case. Then all the feedback will be returned to the algorithm implementation step in order to update the weights of the similarity matrices and consequently improve the performance of the algorithms dynamically. The model is expected to be gradually stabilized and convincing given more feedback is collected and learned.

**Fig. 1:**
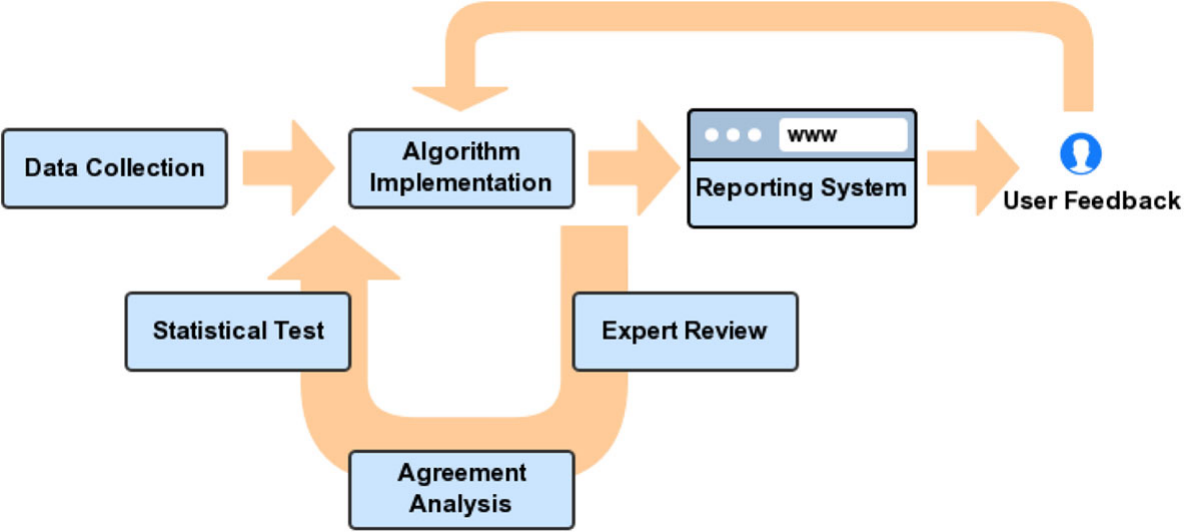
The main steps of workflow for semantic similarity analysis on WebM&M data

The main steps of the workflow are shown in Fig. 1.

#### Case study

In current work, we conducted a detailed study of the three semantic similarity algorithms based on AHRQ PSNet taxonomy. Case 241 [30], a typical event report of nosocomial infection, was chosen as the test query in our study. The similarity list of the query was generated by considering axis “safety target” only, and there were 49 of overall 365 cases with nonzero similarity scores in the result list based on the VS model. The sampling procedure of the 20 cases was as follow. When any of the total 366 cases in the WebM&M database was chosen as a query case, around 50 cases among the other 365 cases had non-zero similarity scores against the query. Considering the draws and close scores, 15 was fixed as an appropriate amount to represent the distribution of the cases with non-zero scores. Then five cases with zero scores were randomly selected and added to the review list because the 4-point Likert scale assumes a quarter of the non-query cases are irrelevant to the query case.

Three domain experts were invited to rate the 20 cases without implication. The result shows that the agreement of the three experts was 90%, and it was more encouraging that the three cases judged as relevant to the query (Case 12 [31], 47 [32], and 336 [33]) had the highest similarity scores calculated by the VS model.

Further analysis on the agreements between algorithms and experts was performed. By comparing to the random model, the VS model and TO model reflect a significantly higher consistency with the experts’ review (Table 2).

**Table 2:** The agreements between the algorithms and the experts

Model	Agreement with Expert 1	Agreement with Expert 2	Agreement with Expert 3
VS	80%**	90%**	90%**
TO	90%**	80%*	90%**
IC	70%	70%	70%

**p-value < 0.01

*0.01 ≤ *p*-value < 0.05

## Discussion

### Data limitations

Here we discuss some of the limitations of WebM&M data. The most obvious defect of WebM&M data is the limited sample size. The inclusion criteria of WebM&M are unclear, however, based on our observation, the cases in WebM&M may be chosen as the most typical ones in each category. For example, patient fall, the most common event type, only has four records in WebM&M, which does not mean patient fall is infrequent, rather indicates the other cases might be similar to the four typical samples and thus were not included by the WebM&M editors. It is also one of the reasons that IC has the worst performance. However, according to the status quo, WebM&M seems to be the best choice for this study since it is by far the only publically accessible taxonomy-based PSE database. Fortunately, the users’ feedback mechanism may reduce the bias caused by the small sample size. The feedback is regarded as an important dynamic parameter which will be used in updating the weights and improving the similarity matrices. Theoretically, the performance of the system would be more effective and reliable as the increase of user feedback.

Another limitation of WebM&M data is the quality of taxonomy. The taxonomy was initially designed for PSE classification and reference rather than providing basic systematic knowledge. In contrast, GO is more suitable for semantic similarity algorithms. Actually, better options, such AHRQ CF, in patient safety community are available with a focus on the structured form only, unfortunately they are not yet linked with WebM&M. Nevertheless, our group is concurrently designing a novel PSE ontology by integrating multiple patient safety resources, based on which new cases reported by users will be automatically annotated. Meanwhile, we are also trying to find a way to make direct connections among different PSE ontologies and annotate previous cases with the new integrated ontology, thereby, there will be a rapid growth in our database. Overall, the assessment of semantic similarity measures in this study is an essential preliminary work. There are limited effects made by the unsatisfied quality of AHRQ PSNet taxonomy.

### Assessment strategy

The assessment of semantic similarity measures on WebM&M data is more challenging than that of GO because there is still no benchmark for the similarity measurement between two PSE. The assessment strategy in this study is based on that the judgments from the experts are considered as gold standard, to which the results of similarity algorithms are compared. Moreover, the agreement ratio can be used to reduce the subjective bias, based on the idea of Content Validity Index [34]. The assessment then turns to a statistical issue regarding testing the mean value of the agreement ratios from semantic similarity approaches and random samplings to expert reviews. Again, as aforementioned, the users’ feedback mechanism is another module designed to complement the lack of assessment methods and to enhance the performance of the whole system.

In the first round of expert review, the 20 cases were selected based on the similarity list provided by VS model. However, it seems unfair to use these cases to measure the performance of the other two models, TO and IC. In order to reduce the assessment biases, additional two rounds of expert reviews would be necessary with a focus on the similarity results of TO and IC respectively.

### The overarching goal

In the field of patient safety, most problems are not just a series of random, unconnected one-off events. Consequently, the basic assumption of our study is that PSE are provoked by weak systems and often have common root causes which can be generalized and corrected. The fundamental role of PSE reporting systems is to enhance patient safety by learning from failures of healthcare system. Thus, we summarized the prototype of an ideal reporting system, representing the objective of our research.

#### Ontology

It is an undoubted kernel of the whole system since an ontology could help us acquire effective experience from previous PSE depending on the quality of the knowledge base. An ideal PSE ontology should be detailed but not cumbersome, and cover all necessary categories and classification criteria of events. The ontology should be able to access the CF and ICPS by providing special interface for format conversation.

#### Database

The size and quality of the database influence the range of knowledge provided by the system. It is always the most difficult part when establishing a database, especially in the patient safety area, the main reasons of which are the absence of public resources and the incompatibility among various data formats.

#### Similarity module

It serves as the engine of the event reporting system since the ultimate goal of using the system, i.e., getting similar cases and potential solutions for the current event, depends on the performance of the similarity algorithms. In terms of the ontology structure, semantic similarity measures seem to be the most applicable approaches for developing the module.

#### Interface

The value of the system is reflected by user experience. Failure in user-centered design may largely account for the issues of low user acceptance and low-quality data that pervasively stored in such PSE systems. A well-designed PSE reporting system should have no limit for multiple educational levels without too much instruction of usage. Besides, as a voluntary reporting system the hints and common solutions should be highlighted since both could motivate users to continue reporting.

## Conclusions

Voluntary PSE reporting systems have great potential for improving patient safety through wide adoption and effective use in healthcare. The similarity analysis of events is a key to the success of such systems. This paper summarized the pros and cons of semantic similarity measures when applicable for comparing PSE, and suggested that two typical approaches effectively serve the comparison purpose. We also provided an initial workflow for applying semantic similarity measures in AHRQ WebM&M data, which are worth particular attention of researchers in the patient safety area. The new generation of PSE reporting system holds promise in triggering a revolution of data management and promoting learning in the patient safety community.

## Abbreviations

AHRQ: The Agency for Healthcare Research and Quality
CF: Common Formats
GO: Gene Ontology
IC: Information Content
ICPS: International Classification of Patient Safety
NTO: Normalized Term Overlap
PSE: Patient Safety Events
PSNet: Patient Safety Network
VS: Vector Space
WebM&M: Morbidity and Mortality Rounds on the Web
